# Cell types and neuronal genetic architecture in the rat CSF-contacting nucleus and the role of 5-HT in this nucleus in mediating morphine addiction through the brain–CSF circuit

**DOI:** 10.3389/fnins.2025.1603486

**Published:** 2025-06-16

**Authors:** Ying Li, Bin Gui, Yijun Zhang, Licai Zhang

**Affiliations:** ^1^Jiangsu Province Key Laboratory of Anesthesiology, Xuzhou Medical University, Xuzhou, China; ^2^Jiangsu Province Key Laboratory of Anesthesiology, and Analgesia Application Technology, Xuzhou Medical University, Xuzhou, China; ^3^NMPA Key Laboratory for Research and Evaluation of Narcotic and Psychotropic Drugs, Xuzhou, China

**Keywords:** the CSF-contacting nucleus, 10x single-cell sequencing, kyoto encyclopedia of genes and genomes, 5-hydroxytryptamine (5-HT), morphine addiction

## Abstract

**Introduction:**

To elucidate the cellular composition of the cerebrospinal fluid-contacting nucleus, establish a comprehensive gene expression database for this nucleus, and investigate its potential functional roles.

**Methods:**

we used single-cell sequencing technology combined with Gene Ontology (GO) and Kyoto Encyclopedia of Genes and Genomes (KEGG) pathway analyses to characterize the transcriptional architecture of the CSF-contacting nucleus and elucidate its potential biological functions. Additionally, Conditioned place preference (CPP) testing, chemogenetic techniques, ELISA, and ultra-performance liquid chromatography-tandem mass spectrometry (UPLC-MS) were employed to examine the functional relationships between the CSF-contacting nucleus, cerebrospinal fluid, and morphine addiction-related behaviors.

**Results:**

Single-cell RNA sequencing revealed that the CSF-contacting nucleus had an average of 22,046 genes expressed per cell. Unsupervised clustering revealed 25 cellular subsets belonging to five canonical CNS cell types (neuron, astrocyte, oligodendrocyte, microglia, endothelial) as annotated against the Genomics RNAseq database. The raw sequencing data have been deposited in the China National Center for Bioinformation (CNCB) accession number: CRR790158. GO and KEGG enrichment analyses demonstrated that the CSF-contacting nucleus neurons were significantly enriched in calcium signaling pathways, neurotransmitter regulation, and addiction-related pathways (including morphine, cocaine, and other substances). Given prior reports of morphine-induced alterations in CSF composition and the unique anatomical features of the CSF-contacting nucleus, we performed additional experimental validation. Chemogenetic manipulation experiments demonstrated that inhibition of the CSF-contacting nucleus attenuated morphine-induced CPP, UPLC-MS and ELISA revealed a marked increase in 5-HT levels in the CSF of the morphine addiction group. Knock out and chemogenetic inhibition of the CSF-contacting nucleus led to a significant reduction in CSF 5-HT levels. These findings suggest that the CSF-contacting nucleus may facilitate morphine addiction through regulated 5-HT release into the CSF. This discovery provides new experimental evidence for understanding CSF-mediated neuromodulation mechanisms.

**Conclusion:**

The present study delineates the single-cell transcriptional architecture and cellular composition of the CSF-contacting nucleus. Bioinformatics analyses revealed the involvement of the CSF-contacting nucleus in multiple addiction-related pathways for various substances of abuse and neurodegenerative disorders. These findings suggest that the CSF-contacting nucleus plays multifaceted roles in neural circuitry, particularly serving as a crucial mediator in neuro-cerebrospinal fluid regulatory mechanisms.

## 1 Introduction

When the cholera toxin B subunit and horseradish peroxidase (CB-HRP) complex, a peripheral nerve tracer, is injected into the lateral ventricle (LV) of an animal, a pair of symmetrical clusters of neurons in the brain parenchyma is consistently labeled, even when the wall of the ventricle is blocked. The terminals of these cerebrospinal fluid (CSF)-contacting neurons extend into the CSF. We verified the existence of the CSF-contacting nucleus in rats, mice and macaques. Because of the invariable location, clustering, and symmetry of the neurons, we call this group of neurons the CSF-contacting nucleus (Song et al., 2019; Song et al., 2020e; Zhou et al., 2013).

The CSF-contacting nucleus is located at the junction of the midbrain and pons, adjacent to the III-VIII cranial nerve nucleus, the dorsal raphe nucleus (DR), and medial longitudinal tract (mlf). The somas of cells in the CSF-contacting nucleus are located in the brain parenchyma and receive extensive projections from the cortex (Song et al., 2020d), the subcortex, the limbic system (Song et al., 2020c), the diencephalon (Song et al., 2020b), the brainstem, and the spinal cord (Song et al., 2020a). The processes of neurons in this nucleus extend into the CSF and both sense and release substances. These neurons play an important role in the bidirectional transmission of information between the brain and CSF (Zhang et al., 2003).

Recently, we reported the transcriptome and functions of the CSF-contacting nucleus (Song et al., 2023). However, the cell types in the CSF-contacting nucleus, the genetic characteristics of the neurons in the nucleus and the mediating of life and disease remain unclear. The development of single-cell sequencing technology in recent years (Armand et al., 2021; Poulin et al., 2016; Tang et al., 2009) has provided an accurate, efficient and high-throughput method to solve the above scientific problems. Here, we used 10x single-cell sequencing technology to first establish a gene database for the CSF-contacting nucleus and identified the cell types in the CSF-contacting nucleus, focusing on the genetic characteristics of neurons, the main cells in the nucleus. The goal was to explore the mechanism by which the CSF-contacting nucleus and 5-HT in this nucleus mediate morphine addiction from a genetic hints.

## 2 Materials and methods

### 2.1 Experimental animals

Specific pathogen-free (SPF)-grade adult male Sprague-Dawley rats weight 250 ± 30 g were obtained from the Laboratory Animal Center of Xuzhou Medical University, [license No. SCXK (Jiangsu) 2015-0009]. All the animal experiments were conducted in accordance with the Guidelines for the Care and Use of Experimental Animals and were approved by the Committee on the Ethical Use of Experimental Animals of Xuzhou Medical University (Ethics Approval number: L20211001001). The animals were housed in controlled environments at temperatures ranging from 23°C to 26°C on a 12 h light/dark cycle with free access to food and water for more than 5 days.

### 2.2 Sample collection

The animals were anesthetized via an intraperitoneal injection of 1% pentobarbital sodium, and the blood was removed by perfusion with saline through the left ventricle. The brain was isolated quickly and placed on ice. According to Song’s empirical formula (Song et al., 2019), an approximately 2,000 μm long piece of brain tissue where the CSF-contacting nucleus is located was obtained. Under direct vision under a 10x stereomicroscope, the cells in the CSF-contacting nucleus were extracted from the ventral gray of the midbrain aqueduct (Aq) and the central gray of the bottom of the fourth ventricle with a Hamilton 12# microinjector (Hamilton, Swiss) and placed in a small liquid nitrogen tank for temporary storage.

### 2.3 Single-cell sequencing and gene database establishment

The samples were resuspended, filtered, screened and purified on a 20 μm screen. Gel beads containing barcode information were combined with a mixture of nuclei and enzymes and coated with oil surfactants to form gel bead in emulsions (GEMs). The GEMs were dissolved, the barcode sequence was released, and the cDNA was reverse transcribed. The cDNA was cut into fragments of approximately 200–300 bp and coupled with sequencing adapters and primers, and PCR amplification was performed using 10x cDNA as a template to construct a standard DNA sequencing library. The constructed library was subjected to 10x single-cell high-throughput sequencing on an Illumina platform in double-ended sequencing mode. After data unloading, a gene expression matrix was obtained, and a gene profile was established using Cell Ranger, the official analysis software of 10x Genomics. A single-cell gene database was established for the CSF-contacting nucleus.

### 2.4 Bioinformatics analysis

(1) Single-cell data analysis of Seurat

The Cell Ranger output was loaded into Seurat^1^ for subsequent cell filtering, normalization, cell subpopulation clustering, differential gene expression analysis across cluster, and marker gene identification.

Seurat employed the following criteria for inter-cluster comparisons:

(a) Genes were expressed in > 10% of cells within either the target cluster or control cluster;
(b) Adjusted *p*-value ≤ 0.01;
(c) Log fold-change (logFC) ≥ 0.26

(2) Differential expression analysis (DEGs)

Differential expression analysis was performed using DESeq2^2^ with the following filtering criteria: FDR < 0.05 and | log2FC| ≥ 1. Genes meeting both criteria were identified as differentially expressed genes (DEGs).

(3) GO, KEGG enrichment analysis

We performed GO enrichment analysis by mapping DEGs to three ontologies (molecular function, cellular component, biological process) in the Gene Ontology database. Gene counts per term were subjected to hypergeometric testing to detect significant enrichment relative to the whole genome. The KEGG enrichment analysis of differentially expressed genes was performed using the KEGG (Kyoto Encyclopedia of Genes and Genomes) database. The KEGG enrichment analysis of DEGs was performed using the KEGG database^3^ focusing on six principal functional categories: cellular processes, organismal systems, metabolism, environmental information processing, genetic information processing, and human diseases. Hypergeometric testing was then employed to identify metabolic pathways that were significantly enriched in the DEGs compared to the whole genomic background. Enrichment results were graphically represented using ggplot2-generated scatter plots in R (version 3.5.2).

This part of the process was performed by LC-Bio Technologies (Hangzhou) Co., Ltd.

### 2.5 Establishment and analysis of a morphine addiction model

Prior to the experiment, animals were placed in a standard conditional place preference box (BHW-DSX1, Anhui, China, AnyMaze video monitoring analysis system, Stoelting, United States) for environmental adaptation and position preference tests, and animals that remained on one side of the box for more than 650 s were excluded.

Included rats were divided into a morphine group (MOP group) and a control group (Ctrl group). The animals in the MOP group were injected with 10 mg/kg morphine hydrochloride (Shenyang Pharmaceutical Company, China) at 8:00 a.m. every day, and they were injected with the same amount of normal saline at 16:00. Animals in the Ctrl group were injected with normal saline via the same method. Each day after injection, the animals were placed in a box with a chamber containing the drug (a) and a chamber not containing the drug (b) for 40 min. The test was repeated for 5 days. Starting on day 6, the addictive behavior of the animals was tested. The percentage of time the animals spent in box a was calculated as follows: time spent in box a/(time spent in box + time spent in box b) × 100%.

If an animal stayed in box a significantly longer than in box b for seven consecutive days, it was considered to exhibit addictive behavior. After morphine was discontinued, there was no significant difference in the time spent by the animals in box a or box b; this phenomenon was considered addiction extinction. After a small dose of morphine (5 mg/kg) was administered to the animals following addiction extinction, a significantly longer time spent in box a than in b box was considered to indicate addiction reinstatement (Wang et al., 2020).

### 2.6 Specific targeting of the CSF-contacting nucleus

For chemogenetic activation of the CSF-contacting nucleus (Ga group), 200 nl recombinant adeno-associated virus rAAV2/9-hSyn-DIO-hM3Dq-mCherry (BrainCase, Shenzhen, China) was injected into the CSF-contacting nucleus (coordinates were selected according to Song and Zhang’s empirical formula) (Song et al., 2019). A total of 1,000 nl recombinant adeno-associated virus rAAV2/9-hSyn-SV40 LS-Cre (BrainCase, Shenzhen, China) was simultaneously injected into the left ventricle.

For chemogenetic inhibition of the CSF-contacting nucleus (Gi group), 200 nl recombinant adeno-associated virus rAAV2/9-hSyn-DIO-hM4Di-EGFP (BrainCase, Shenzhen, China) was injected into the CSF-contacting nucleus in the same manner as for chemogenetic activation.

In the Ctrl group, the recombinant adeno-associated virus rAA2/9-hSyn-DIO-EGFP, which was used to activate or inhibit the promoter, was not injected into the CSF-contacting nucleus; however, the recombinant adeno-associated virus rAAV2/9-hSyn-SV40 NLS-Cre was injected into the lateral ventricle. The injection coordinates and dosage were the same as those used for the Ga and Gi groups.

The animals were fed for 21 days and killed at the end of all tests. Brain tissue containing the CSF-contacting nucleus was obtained, and coronal sections were prepared. The recombinant adeno-associated virus in the CSF-contacting nucleus was detected via immunofluorescence, and positive signals were observed, photographed and analyzed via laser confocal microscopy. After the end of the experiment, only the data for animals with good expression of the virus in the CSF-contacting nucleus were included, and data for the other animals were excluded.

To “knockout” the CSF-contacting nucleus, as described by Song and Zhang (2018), a peripheral nerve-destroying agent, cholera toxin B subunit combined with saponin (CB-SAP), was injected into the LV of the animals, and the animals were maintained for 7 days. After the experiment was completed, coronal sections of brain tissue containing the CSF-contacting nucleus were prepared. Immunofluorescence staining was used to visualize residual neurons in the CSF-contacting nucleus, and laser confocal microscopy was used to count and image them; statistical analysis of the experimental data was performed to verify the complete destruction of the CSF-contacting nucleus.

### 2.7 Immunofluorescence (IF)

Brain tissue containing the CSF-contacting nucleus was collection, and frozen coronal sections with a thickness of 40 μm were prepared. Immunofluorescence (IF) staining was performed with antibodies against cholera toxin subunit B (CB-594) (Thermo Fisher, United States, Cat# 34777) and c-Fos (Cell Signaling Technology, United States Cat# 2250S) and a goat anti-serotonin polyclonal antibody (Abcam, United States, Cat# ab66047). The samples were washed, dried, and sealed, and a confocal laser scanning microscope (FV1000, Olympus, Japan) was used for observation, imaging, and counting.

### 2.8 Statistics

SPSS 16.0 software was used for statistical analysis. The data are expressed as the means ± SD or means ± SE. Student’s *t*-test or the Mann-Whitney test was used for between-group comparisons, as appropriate. One-way ANOVA followed by the Student-Newman-Keuls *post hoc* test was used for comparisons among different groups. Two-way ANOVA with repeated measurements was used to test the behavioral results at different time points followed by Tukey’s *post hoc* test. GraphPad Prism 7.0 software was used to generate graphs. *P*-value < 0.05 was considered significant.

## 3 Results

The total number of genes expressed in the CSF-contacting nucleus was 22046. All the data were submitted to the China National Biological Information Center (CNCB) - National Genomics Data Center (NGDC) (GSA number: CRA011168).

### 3.1 There are five types of cells in the CSF-contacting nucleus

**FIGURE 1:**
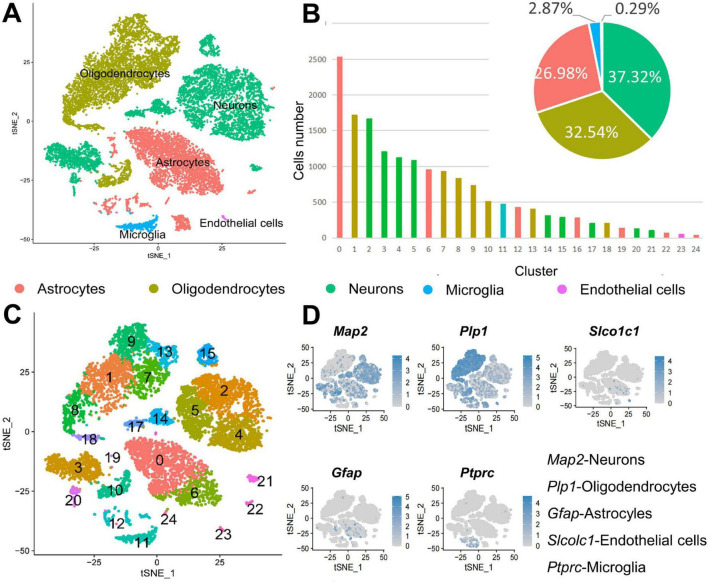
Cell types in the cerebrospinal fluid (CSF)-contacting nucleus. **(A)** t-SNE diagram of the five cell types in the CSF-contacting nucleus. **(B)** Distribution of the five types of cells in the 25 clusters and their proportions relative to all cells. Expression of marker genes of the five cell types in the CSF-contacting nucleus. **(C)** t-SNE diagram of the expression of marker genes of the five cell types in the 25 clusters. **(D)** Expression of marker genes of the five cell types in the CSF-contacting nucleus (Supplementary Figure 1).

Gene expression in cells in each sample was compared with that in 18 known cell type to identify the cell types in the CSF-contacting nucleus, and the results were further validated by analyzing the expression of known cellular markers. Dimensionality reduction and clustering were performed for the gene expression data for the CSF-contacting nucleus were processed to determine the distribution of different cell types in all clusters. Neurons, astrocytes, oligodendrocytes, microglia, and vascular endothelial cells are present in the CSF-contacting nucleus. Neurons comprised clusters 2, 3, 4, 5, 14, 15, 17, 20, and 21. Oligodendrocytes comprised clusters 1, 7, 8, 9, 10, 13, and 18. Astrocytes comprised clusters 0, 6, 12, 16, 19, 22, and 24. Microglia comprised only cluster 11. Vascular endothelial cells comprised only cluster 23. Figures 1A, D. There were 6,108 neurons in the CSF-contacting nucleus, accounting for 37.32% of the total number of cells and ranking first among cell types in terms of abundance. There were 5,325 oligodendrocytes, accounting for 32.54% of all cells and ranking second among in terms of abundance. There were a total of 4,416 astrocytes, accounting for 26.98% of all cells and ranking third in terms of abundance. There were 469 microglia (2.87%) and 47 vascular endothelial cells (0.029%) (Figures 1B, C).

### 3.2 The top 10 DEGs in each neuronal cluster

**FIGURE 2:**
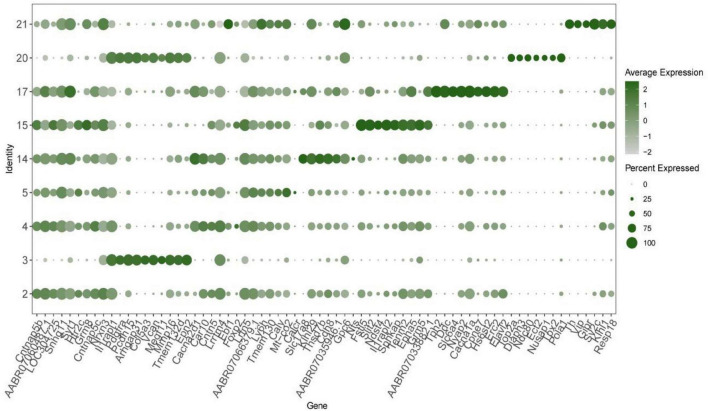
The top 10 differential expression analysis (DEGs) in each neuronal cluster in the cerebrospinal fluid (CSF)-contacting nucleus (duplicate genes were removed). Verification of computational method.

The 90 top differential expression analysis (DEGs) in the nine neuronal clusters (i.e., the top 10 genes for each cluster) were analyzed. Since 31 genes were repeated, the functions of a total of 69 genes were analyzed. Among them, 16 genes were found to be involved in calcium ion regulation. Genes related to the synthesis and release of 5-HT and other transmitters, such as *Th*, *Tph2* and *Htr2c*, were highly expressed. The synapse binding protein I (*Syt1*) gene was expressed in clusters 2, 14, 17, and 21. The tyrosine kinase type III receptor and GDP molecular switch gene *Arhgap31* ere expressed in clusters 3 and 20. Genes related to GABA receptors and cAMP-G signaling pathway regulation were also among the top 10 genes in each cluster. Notably, the *KLHL29* gene, which is associated with Bardet–Biedl syndrome in humans, and *Pcdh15*, which is associated with Usher syndrome, were amongthe top 10 DEGs (Figure 2).

### 3.3 Gene ontology (GO) enrichment analysis of the top 20 DEGs in the neuronal clusters in the CSF-contacting nucleus

**FIGURE 3:**
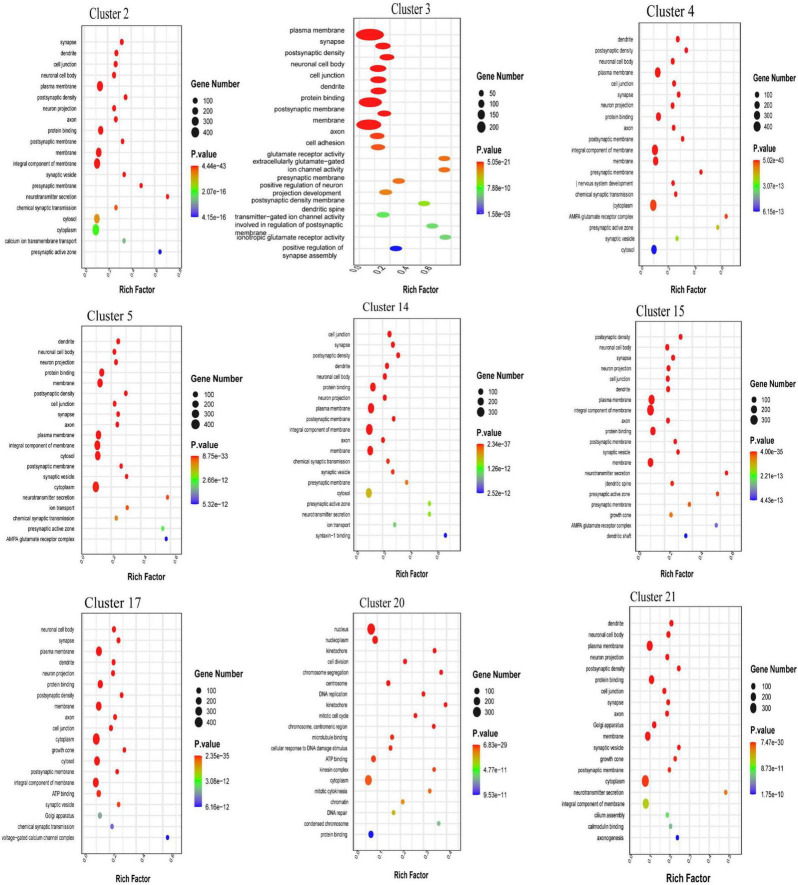
Gene ontology (GO) enrichment analysis of the the top 20 differential expression analysis (DEGs) in each neuronal cluster in the cerebrospinal fluid (CSF)-contacting nucleus (Supplementary Figure 3).

The biological process terms in which the DEGs in cluster 20 were enriched included ion transmembrane transport, signal transduction, DNA and RNA transcription, protein phosphorylation, chemical synaptic transmission, redox regulation and cell differentiation regulation. In particular, the DEGs were found to be related to apoptosis, the response to drugs, and nerve, heart and multicellular organ development. The DEGs in clusters 2, 4, 5, 17, and 21 were found to be associated with the cell component terms Golgi apparatus, mitochondria and dendrites, and DEGs in almost every neuronal cluster were enriched in the terms neuronal projection and exosome. The main molecular function terms in which the DEGs in clusters 3, 14, and 15 were enriched were ATP, RAN, DNA and protease binding, especially calcium ion, zinc ion and dendritic protein binding (Figure 3).

### 3.4 Kyoto encyclopedia of genes and genomes (KEGG) enrichment analysis of the top 5 DEGs in each neuronal cluster in the CSF-contacting nucleus

**FIGURE 4:**
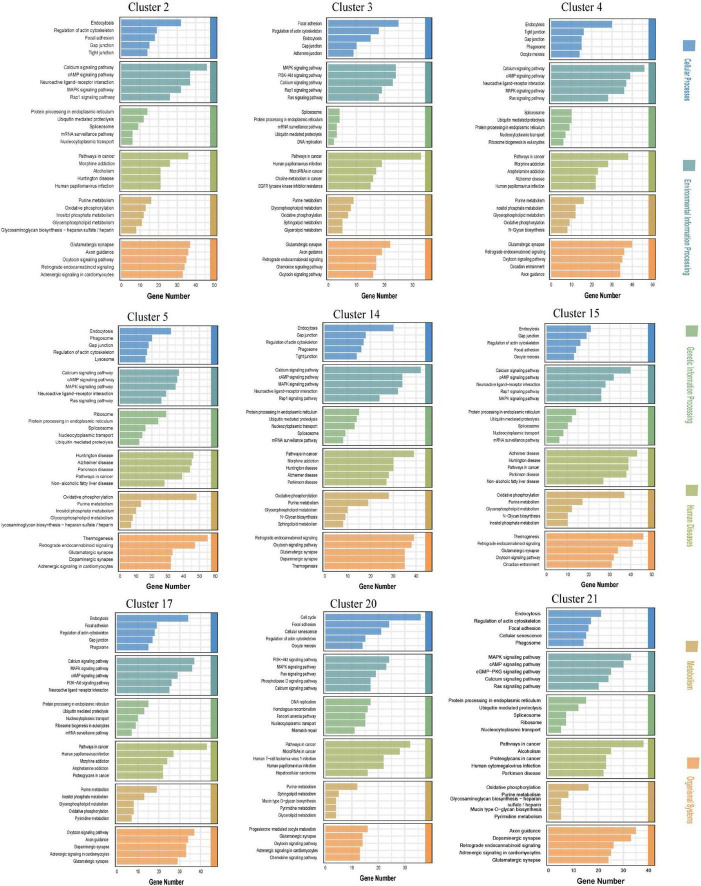
Kyoto encyclopedia of genes and genomes (KEGG) enrichment analysis of the top five differential expression analysis (DEGs) in the neuronal clusters in the cerebrospinal fluid (CSF)-contacting nucleus (Supplementary Figures 2, 4–12).

The main human diseases in which the DEGs were enriched were Alzheimer’s disease, Parkinson’s disease, Huntington’s disease, morphine addiction, nicotine addiction, methamphetamine addiction, alcoholism, non-alcoholic fatty liver disease, human T-cell leukemia virus type 1 infection, human papillomavirus infection, human cytomegalovirus infection, cancer micrornas, cancer choline metabolism, cancer proteoglycans, hepatocellular carcinoma, etc. The main cellular processes in which the DEGs were enriched were endocytosis, focal adhesion, gap junction, tight junction, and actin cytoskeleton regulation. The main environmental information processing pathways in which the DEGs were enriched were the calcium signaling pathway, the cAMP signaling pathway, the MAPK signaling pathway, the Rap1 signaling pathway, the PI3K-Art signaling pathway, the Ras signaling pathway, the neural activity ligand-receptor interaction pathway and other related pathways. The main genetic information processing pathways in which the DEGs were enriched were endoplasmic reticulum protein processing, the ubiquitin-mediated protein degradation system, DNA replication, nucleoplasmic exchange, homologous recombination, mismatch repair, the Fanconi anemia pathway, etc. The main metabolism-related pathways in which the DEGs were enriched were oxidative phosphorylation, purine metabolism, pyrimidine metabolism, sphingolipid metabolism, glycerol metabolism and inositol phosphate metabolism. The main organismal system pathways in which the DEGs were enriched were axon guidance, dopaminergic synapses, glutamatergic synapses, retrograde endocannabinoid signaling, intracellular adrenergic signaling, circadian rhythm interference, etc., (Figure 4).

### 3.5 Neuronal activity in the CSF-contacting nucleus and changes in the levels of substances in the CSF of morphine-addicted rats

**FIGURE 5:**
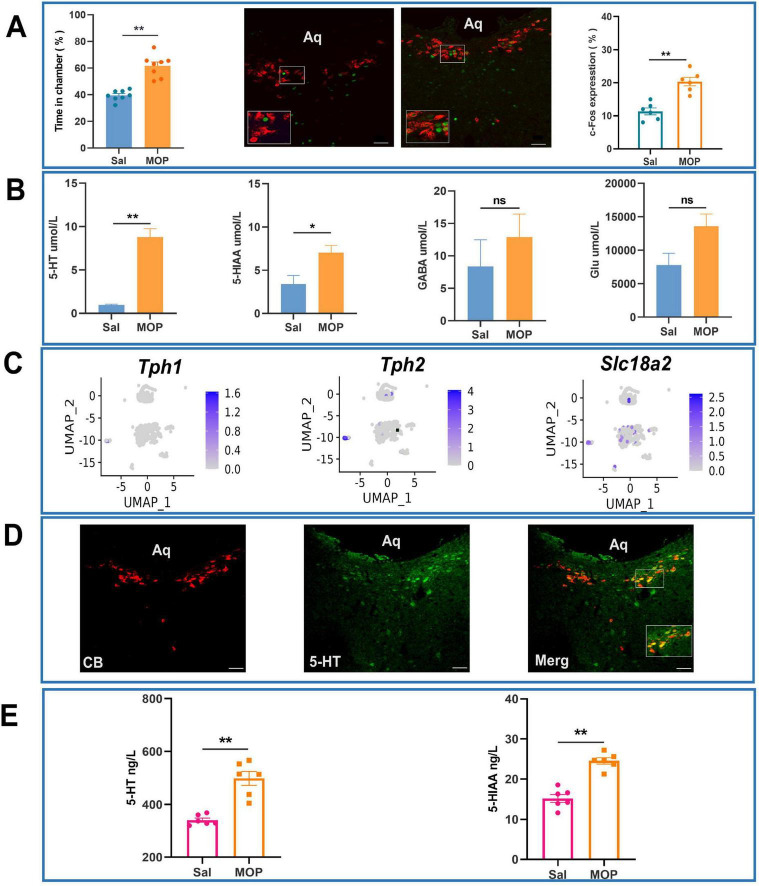
Morphine addiction alters cerebrospinal fluid (CSF) 5-hydroxytryptamine (5-HT) dynamics and neuronal activation patterns in the CSF-contacting nucleus of rats. **(A)** Immunofluorescence analysis of c-Fos expression in Sal and MOP groups with statistical validation. **(B)** Effects of morphine addiction on the levels of substances in the CSF of rats (according to UPLC-MS/MS). **(C)** Distribution of 5-HT-related genes in neuronal clusters in the CSF-contacting nucleus (t-SNE diagram). **(D)** Expression of 5-HT (green) in the CSF-contacting nucleus (red, inside the dashed ellipse) (IF image). Bar = 50 μm. **(E)** Effects of morphine addiction on 5-HT and 5-HIAA levels in the CSF of rats (as determined by ELISA). **P < 0.01, *P < 0.05. *n* = 6.

Compared with those in the normal saline group (Sal group), the rats in the morphine addiction group (MOP group) presented obvious CPP, and the time spent (%) in the chamber containing the drug was much longer than that in the chamber not containing the drug. c-Fos expression in the CSF-contacting nucleus was much greater in the MOP group than in the Sal group (*P* < 0.01) (Figure 5A).

Ultra-performance liquid chromatography-tandem mass spectrometry (UPLC-MS) revealed that the level of 5-HT in the CSF of rats in the MOP group was significantly increased compared with that in the CSF of rats in the Sal group (P < 0.01). The level of 5-HIAA, a metabolite of 5-HT, in the CSF of rats in the MOP group was increased compared with that in the CSF of rats in the Sal group (*P* < 0.05) (Figure 5B).

Uniform Manifold Approximation and Projection (UMAP) visualization of 5-HT-related marker gene distribution further revealed widespread 5-HT expression across neuronal clusters within the CSF-contacting nucleus (Figure 5C). Immunofluorescence assays revealed that almost all of the neurons in the CSF-contacting nucleus were 5-HTergic neurons (Figure 5D).

Enzyme-linked immunosorbent assay (ELISA) was performed to further verify that the levels of 5-HT and its metabolite 5HIAA in the CSF of rats in the MOP group were significantly greater than those in the CSF of rats in the Sal group (*P* < 0.01) (Figure 5E).

### 3.6 Changes in addictive behavior and 5-HT levels in the CSF after chemogenetic activation or inhibition of the CSF-contacting nucleus

**FIGURE 6:**
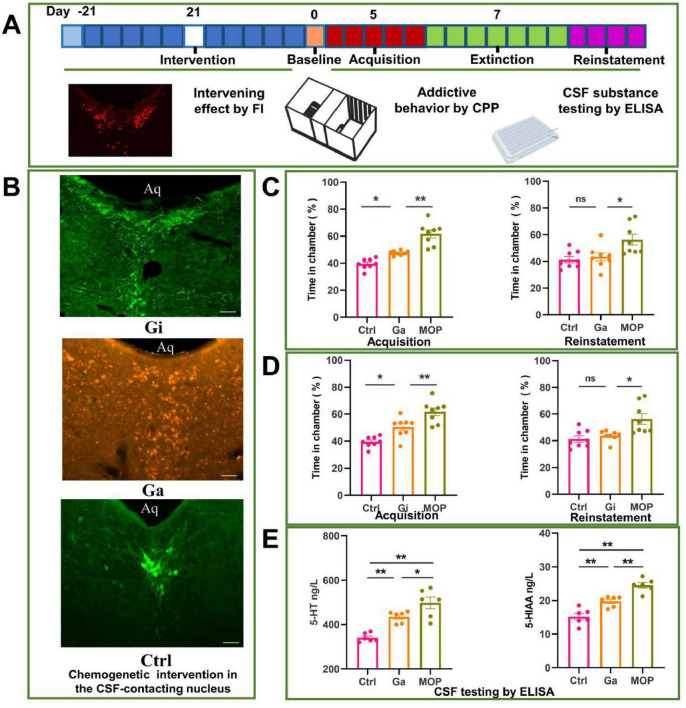
Effect of chemogenetic activation and inhibition of the cerebrospinal fluid (CSF)-contacting nucleus on conditioned position preference (CPP) behavior and the levels of substances in the CSF of rats. **(A)** Flow chart of the experiment. **(B)** Image of the adeno-associated virus infecting the CSF-contacting nucleus. **(C)** Effects of chemogenetic activation of the CSF-contacting nucleus on CPP following the development and reinstatement of morphine addiction. **(D)** Effects of the chemogenetic inhibition of the CSF-contacting nucleus on CPP after the development and reinstatement of morphine addiction. **(E)** Effect of the chemogenetic activation of the CSF-contacting nucleus on the levels of 5-hydroxytryptamine (5-HT) and 5-HIAA in the CSF (as determined by ELISA). Bar = 50 μm. **P < 0.01, *P < 0.05. *n* = 8.

The experimental process used for the chemogenetics experiment and the parameters observed are shown in Figure 6A. Immunofluorescence images of brain sections showing evidence of chemogenetic activation (Ga group) and chemogenetic inhibition (Gi group) of the CSF-contacting nucleus and the control group (Ctrl group) are shown in Figure 6B. The CSF-contacting nucleus was completely infected by the adeno-associated virus. The development of addiction was promoted in the Ga group, and preference for the drug-containing chamber in the Ga group was significantly greater than that in the Ctrl group (*P* < 0.05). However, the preference for the drug-containing chamber in the Ga group was not as high as that in the MOP group, and the difference was significant (*P* < 0.01). The Ga reinstatement CPP after extinction was not significantly different from that of the Ctrl group, but significantly lower than that of the MOP group (*P* < 0.05) (Figure 6C).

The development of morphine addiction was significantly inhibited in the Gi group; the time spent in the drug-containing chamber (%) in the Gi group was significantly lower than that in the MOP group (*P* < 0.01), but it did not reach the normal level (*P* < 0.01) (Figure 6D).

Chemogenetic inhibition of the CS-contacting nucleus inhibited the reinstatement of morphine addiction in rats after extinction. Reinstatement behavior was alleviated in the Gi group compared with the MOP group, the time spent in the drug-containing chamber was increased in the Gi group compared with the Ctrl group (*P* < 0.05), although the difference was not statistically significant.

The level of 5-HT and its metabolite 5-HIAA in the CSF was increased in the Ga group compared with the Ctrl group (*P* < 0.05), but it did not reach that high than the MOP group (Figure 6E).

### 3.7 Changes in the addictive behavior of rats with the CSF-contacting nucleus knockout after infusion of 5-HT into the CSF

**FIGURE 7:**
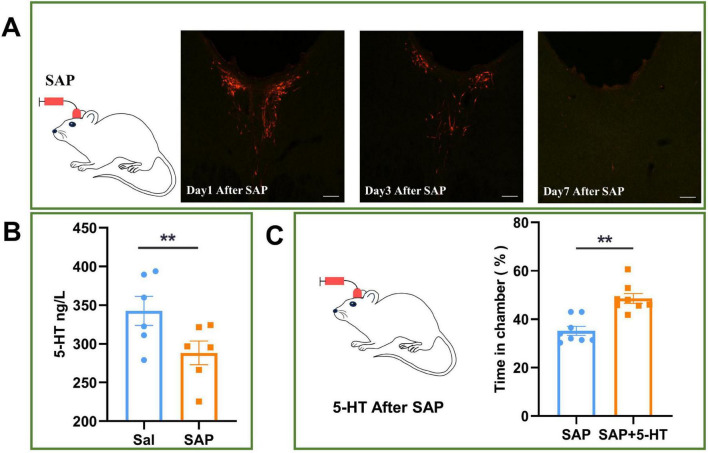
Effect of 5-hydroxytryptamine (5-HT) infusion into cerebrospinal fluid (CSF) on conditioned position preference (CPP) in rats with CSF-contacting nucleus knockout. **(A)** After 7 days of infusion of saponin (SAP) into the left ventricle, the CSF-contacting nucleus was knocked out. **(B)** Effect of knocking out the CSF-contacting nucleus on the level of 5-HT in the CSF of rats. **(C)** Effect of 5-HT infusion into the CSF on CPP in rats with the CSF-contacting nucleus knockout. Bar = 50 μm. **P < 0.01. *n* = 6.

The neuron-destroying agent SAP was infused through the LV for 7 days to specifically destroy (knock out) the CSF-contacting nucleus. After establishment of the CSF-contacting nucleus knockout model, 5-HT levels in the CSF of the model animals were significantly lower than those in the CSF of animals in the saline group (*P* < 0.05) (Figures 7A, B).

Then, 5-HT was infused into the left ventricle to achieve a 5-HT level equal to that before knockout of the CSF-contacting nucleus (SAP + 5-HT group), and addictive behavior was significantly recovered in the SAP + 5-HT group compared with the group in which the CSF-contacting nucleus was knocked out but 5-HT was not infused into the left ventricle (SAP group) (*P* < 0.05) (Figure 7C).

## 4 Conclusion

In this study, single-cell RNA sequencing combined with validated cell-specific marker analysis revealed that the CSF-contacting nucleus comprises five major cell types, further classified into 25 clusters. Among these, neurons constituted nine clusters (37.32% of total cells), oligodendrocytes seven clusters (32.54%), astrocytes seven clusters (26.98%), microglia one cluster (2.87%), and endothelial cells one cluster (0.029%). Neurons were the most abundant cells in the CSF-contacting nucleus, with neurons accounting for nine out of 25 cell clusters, as shown in Figure 1. Thus, we focused on the transcriptome of neurons. We studied the top 10 DEGs in the nine neuronal clusters (i.e., 90 DEGs) and found that 16 of the DEGs in neurons in the CSF-contacting nucleus were related to calcium channel regulation; moreover, there were many DEGs related to the regulation of acetylcholine, 5-hydroxyserotonin, GABA, the sodium threshold, etc. Notably, some of the DEGs were associated with Bardet–Biedl syndrome and Usher syndrome (Figure 2). These results suggest that neurons in the CSF-contacting nucleus have excellent calcium regulation ability, are rich in 5-HT and GABA receptors, and may play a role in human Bardet–Biedl syndrome and Usher syndrome.

Kyoto encyclopedia of genes and genomes and GO analyses collectively delineated the general functional characteristics of the CSF-contacting nucleus. In addition to common neuronal gene signatures, we observed significant enrichment of projection regulation and extracellular vesicle-related pathway genes in the cerebrospinal fluid-contacting nucleus, with extracellular vesicle genes being ubiquitously expressed across all nine neuronal clusters. Notably, KEGG human disease pathways implicated the CSF-contacting nucleus neurons in multiple substance addiction circuits, including morphine, amphetamine, nicotine, and cocaine dependence, as well as neurodegenerative disorders.

Functional validation through c-Fos immunofluorescence and chemogenetic manipulation confirmed the CSF-contacting nucleus neuronal activation during morphine-induced CPP. Given the unique anatomical positioning of the CSF-contacting nucleus, we further investigated its role in morphine addiction by integrating UPLC-MS, ELISA, immunofluorescence staining with behavioral assays. These experimental results identified the role of a specific neuronal population in the CSF-contacting nucleus the serotonergic neurons in morphine addiction. Specifically, the findings demonstrate that the CSF-contacting nucleus may contribute to the formation of morphine addiction through the release of 5-HT by its serotonergic neurons into the CSF.

Collectively, these findings provide novel insights into the tripartite interplay between the CSF-contacting nucleus, morphine addiction, and CSF signaling mechanisms.

## 5 Discussion

Although clinicians have long diagnosed diseases by detecting changes in the levels of substances in the CSF to diagnose diseases and treated diseases by infusing drugs or even cells into the CSF (Nelson-Maney et al., 2024; Orlovska-Waast et al., 2021; Wong et al., 2023), the source of substances in CSF and the anatomical basis for their action via the CSF pathway are not clear. The discovery of the CSF-contacting nucleus not only provides a clear anatomical basis for brain-CSF interactions but also provides a new avenue for elucidating the mechanisms that regulate vital biological functions and diseases involving the CSF pathway. Therefore, investigating the biological characteristics of the CSF-contacting nucleus as soon as possible is particularly important.

Genes are the source of life activities and diseases, and gene annotation is one of the best methods for predicting the physiological function of cells (Angeloni and Bogdanovic, 2019; Harry and Zakas, 2024). In this study, we first established a gene database for the CSF-contacting nucleus. By comparing the genes expressed by cells in the CSF-contacting nucleus with those known to be expressed by different cell type, we confirmed for the first time that the CSF-contacting nucleus contains neurons, astrocytes, oligodendrocytes, microglia and vascular endothelial cells.

Gene ontology enrichment analysis of the top 20 DEGs in the nine neuronal clusters revealed that the neurons in the CSF-contacting nucleus were similar to ordinary neurons in terms of biological processes, cellular components and molecular functions and were also associated with other biological functions, such as heart, brain and multicellular organ development; the drug response; calcium ion regulation; and fiber projections, secretion and release (Figure 3).

Kyoto encyclopedia of genes and genomes enrichment analysis is a common method for studying the advanced functions of cells (Kanehisa and Sato, 2020; Kanehisa et al., 2008). Our findings suggested that the cellular processes, environmental information processing, and metabolism related to the genes expressed by neurons in the CSF-contacting nucleus are similar to those related to the genes expressed by ordinary neurons. However, in terms of genetic information processing, organismal systems, and human disease, compared with ordinary neurons, neurons in the CSF-contacting nucleus may play a more specific role in the regulation of hereditary diseases such as Fanconi anemia (Gillio et al., 1997), retroactive endocannabinoid signaling, circadian disturbances, neurodegenerative diseases such as Alzheimer’s disease (Liu et al., 2021), addiction to drugs such as morphine (Herrero-Turrión et al., 2014), non-alcoholic fatty liver disease (Mahmoudi et al., 2022), many infections (Slizen and Galzitskaya, 2020), and cancer (Zhang et al., 2021; Figure 4).

This study revealed that the neurons in the CSF-contacting nucleus are rich in 5-HT (Figure 5). This further supports our previous findings that more than 96% of the cells in the CSF-contacting nucleus contain 5-HT (Xing et al., 2015; Zhai et al., 2023). In another study, we demonstrated that damaging the CSF-contacting nucleus significantly reduces morphine withdrawal symptoms in rats (Lu et al., 2010; Lu and Zhang, 2011; Qing et al., 2007). This study further revealed that neuronal activity in the CSF-contacting nucleus was significantly increased and that the level of 5-HT in the CSF was significantly increased, as shown by UPLC-MS/MS and ELISA, in morphine-addicted rats (Figure 5). The chemogenetic activation and inhibition of the CSF-contacting nucleus affected CPP in morphine-addicted animals to varying degrees, and the level of 5-HT in the CSF changed accordingly (Figure 6). CPP could be restored and enhanced by infusion of an adequate amount of 5-HT into the CSF after knockout of the CSF-contacting nucleus. These results suggest that the CSF-contacting nucleus and 5-HT in this nucleus may mediate morphine addiction through the CSF.

Hydroxytryptamine is one of the most important transmitters in the nervous system and is widely involved in various physiological and pathological processes in the body (Hashemi et al., 2012). Many studies have shown that 5-HT plays a role in addiction to multiple drugs (Li et al., 2011). To date, the mechanism of addiction remains an open question. Previous studies on addiction have focused on neural circuits (Chen et al., 2022), receptors and signaling pathways (D’Addario et al., 2014; Mahler et al., 2019), synaptic plasticity (Jones and Bonci, 2005), and many other factors. However, these studies focused primarily on communication among neurons and neglected the role of body fluids, especially the CSF, in addiction.

The CSF-contacting nucleus emerges as a promising therapeutic target for drug addiction interventions. Potential strategies could include modulating 5-hydroxytryptamine (5-HT) release or blocking CSF-contacting nucleus activity to develop innovative anti-addiction treatments. Furthermore, dynamic monitoring of CSF 5-HT levels may serve as a valuable biomarker for assessing addiction severity or treatment efficacy. Future research should employ spatial transcriptomics and single-cell multi-omics technologies to further elucidate neuronal subpopulation heterogeneity and functional specialization within the CSF-contacting nucleus. Additionally, investigation of downstream targets for 5-HT released by the CSF-contacting nucleus (such as specific brain regions or receptors) will be crucial for understanding how this nucleus transmits signals via CSF to influence addiction-related neural circuits.

## Data Availability

The datasets presented in this study can be found in online repositories. The names of the repository/repositories and accession number(s) can be found below: ftp://download.big.ac.cn/gsa2/CRA011168, CRA011168.

## Footnotes

1
https://satijalab.org/seurat/

2
http://bioconductor.org/packages/stats/bioc/DESeq2/

3
www.genome.jp/kegg
