# Supplementary material for: Cell types and neuronal genetic architecture in the rat CSF-contacting nucleus and the role of 5-HT in this nucleus in mediating morphine addiction through the brain–CSF circuit
Source: Front Neurosci. 2025 Jun 16;19:1603486. doi: 10.3389/fnins.2025.1603486 (PMC12206836; doi:10.3389/fnins.2025.1603486)
Supplement: Supplementary file 1 [file Data_Sheet_1.pdf]

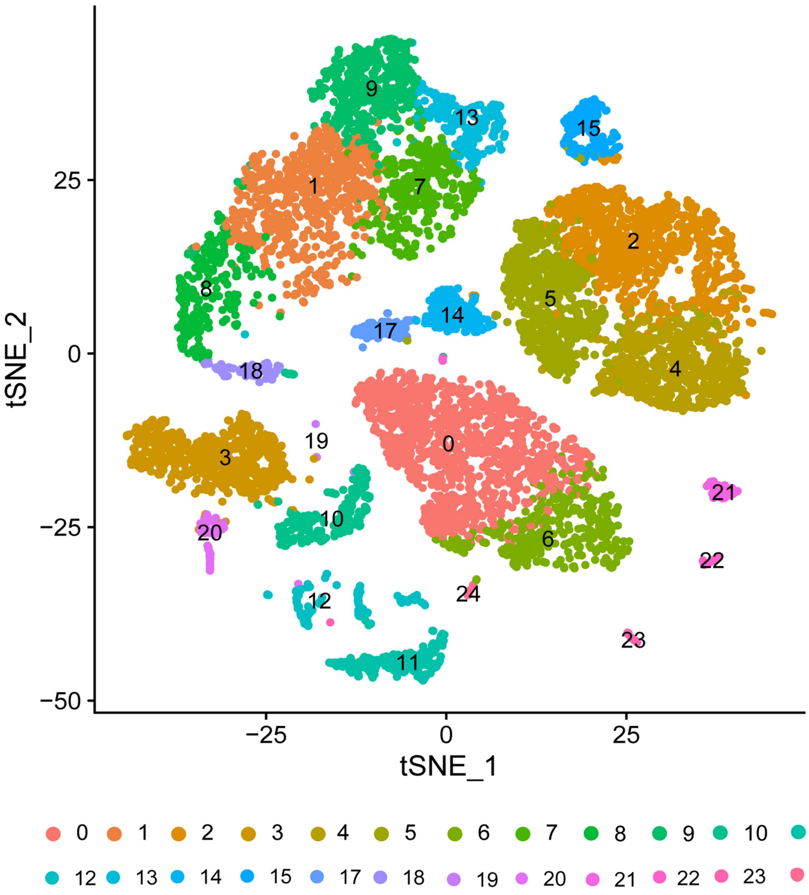


Supplementary Figure 1: t-SNE diagram displays the distribution of 25 transcriptionally distinct clusters.

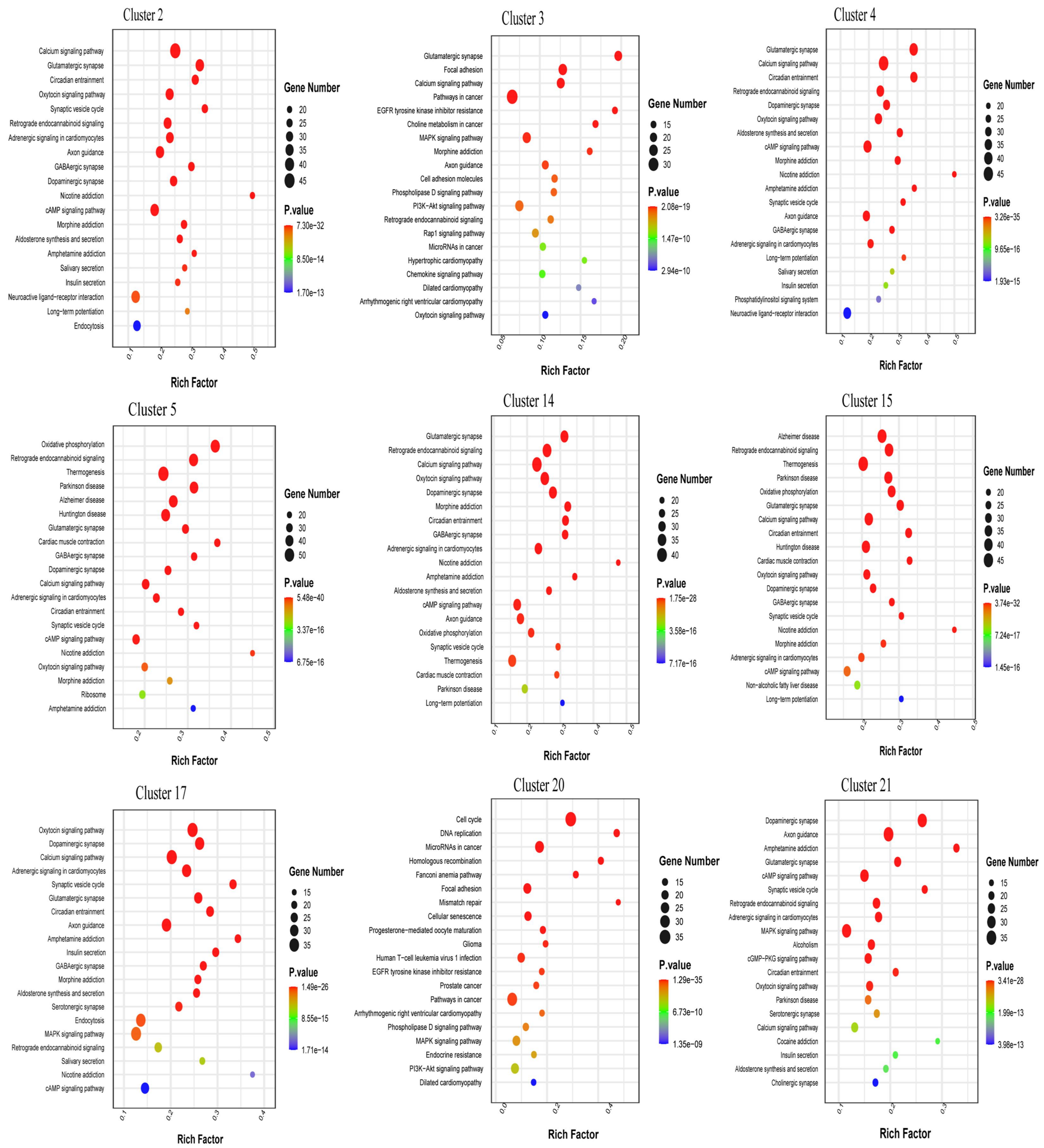

Supplementary Figure 2: Neural cluster representative differential expressed gene KEGG_enrichment analysis

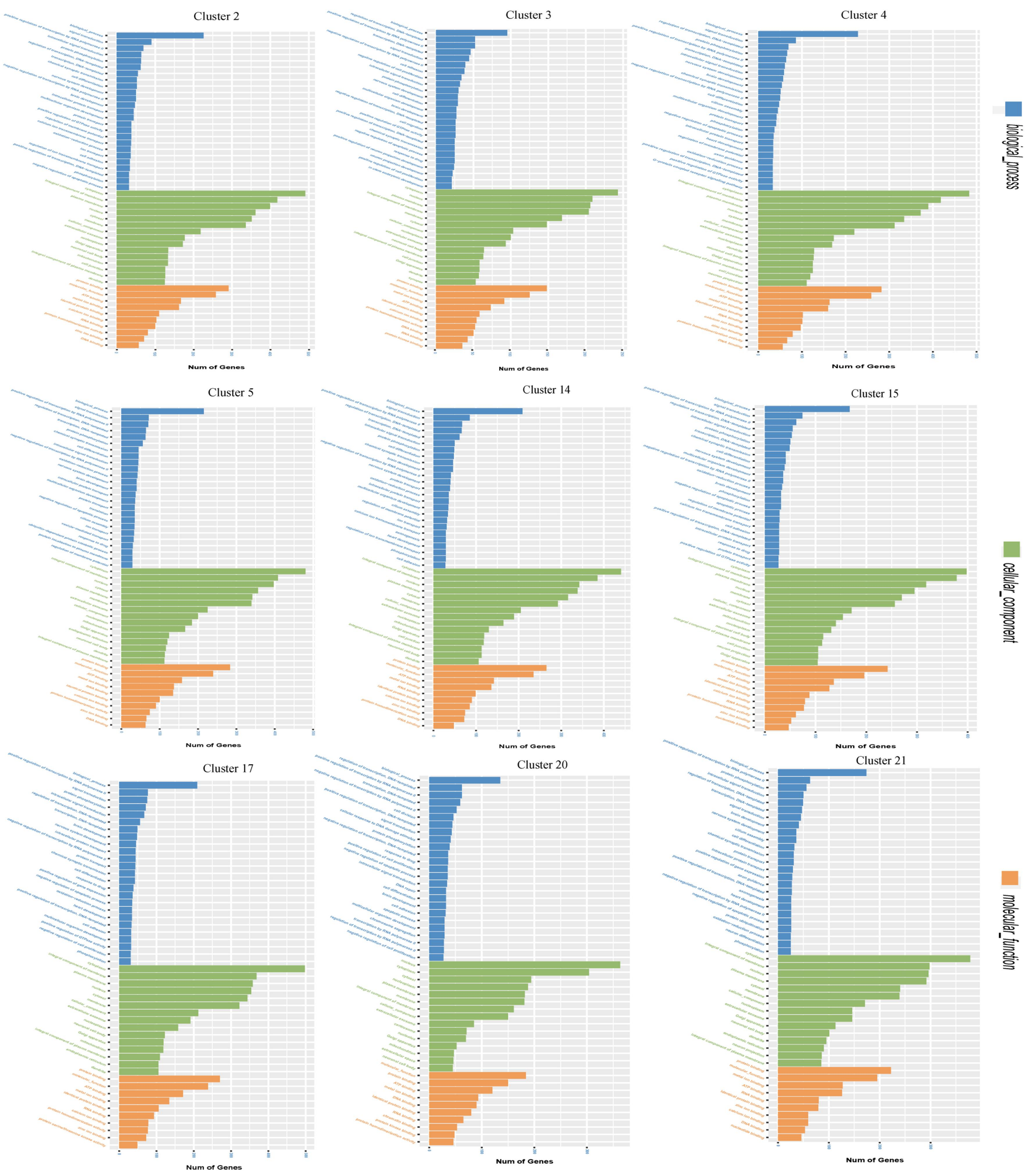


Supplementary Figure 3: Neural cluster representative differential expressed gene GO_enrichment analysis.

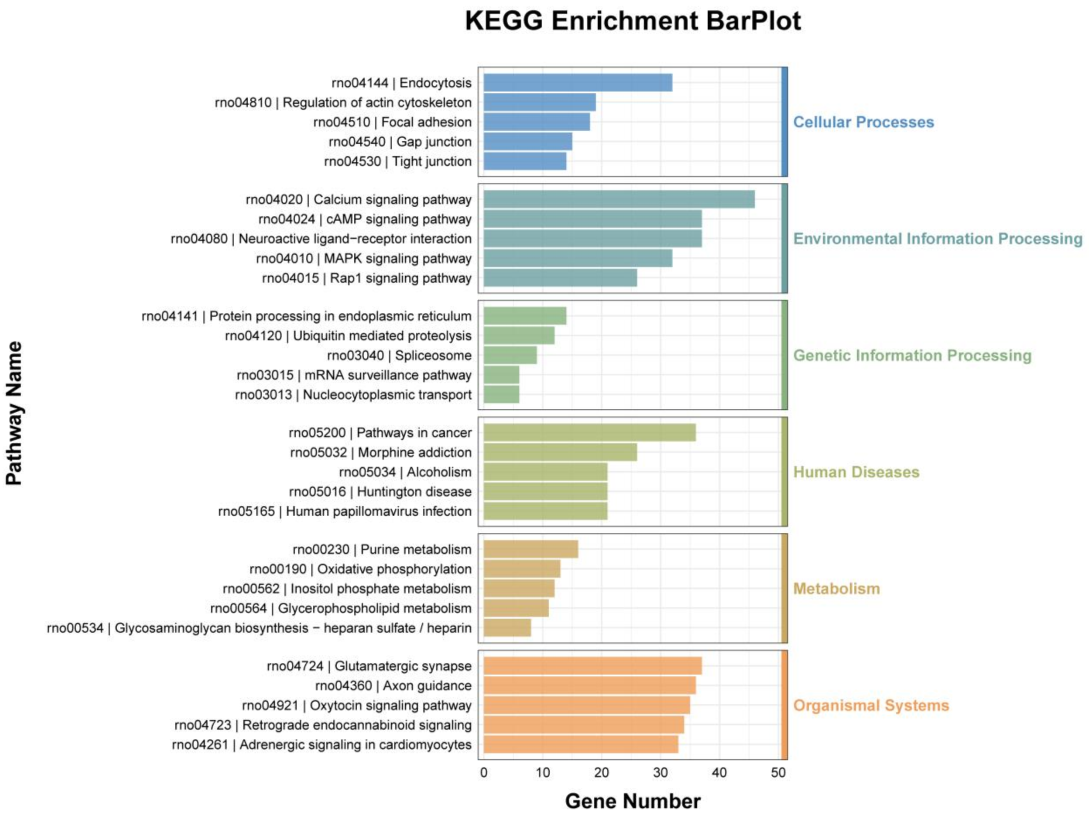


Supplementary Figure 4: KEGG enrichment analysis in the neuronal 2 clusters

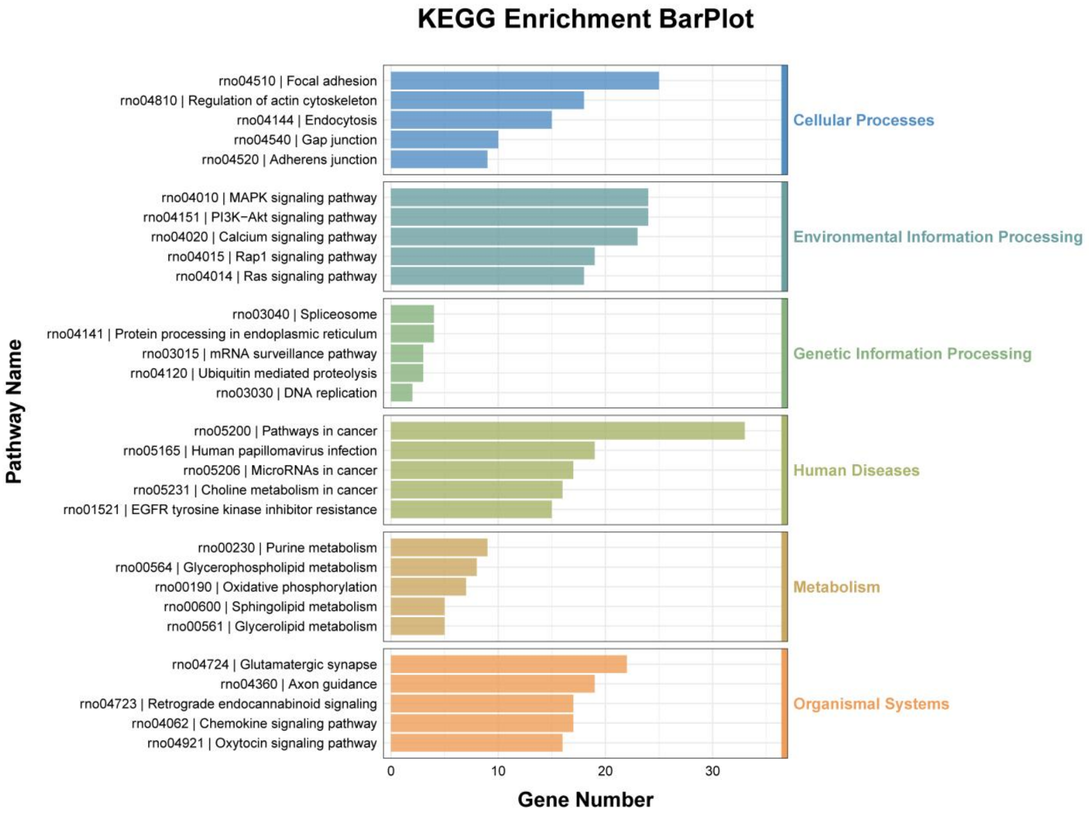


Supplementary Figure 5: KEGG enrichment analysis in the neuronal 3 clusters

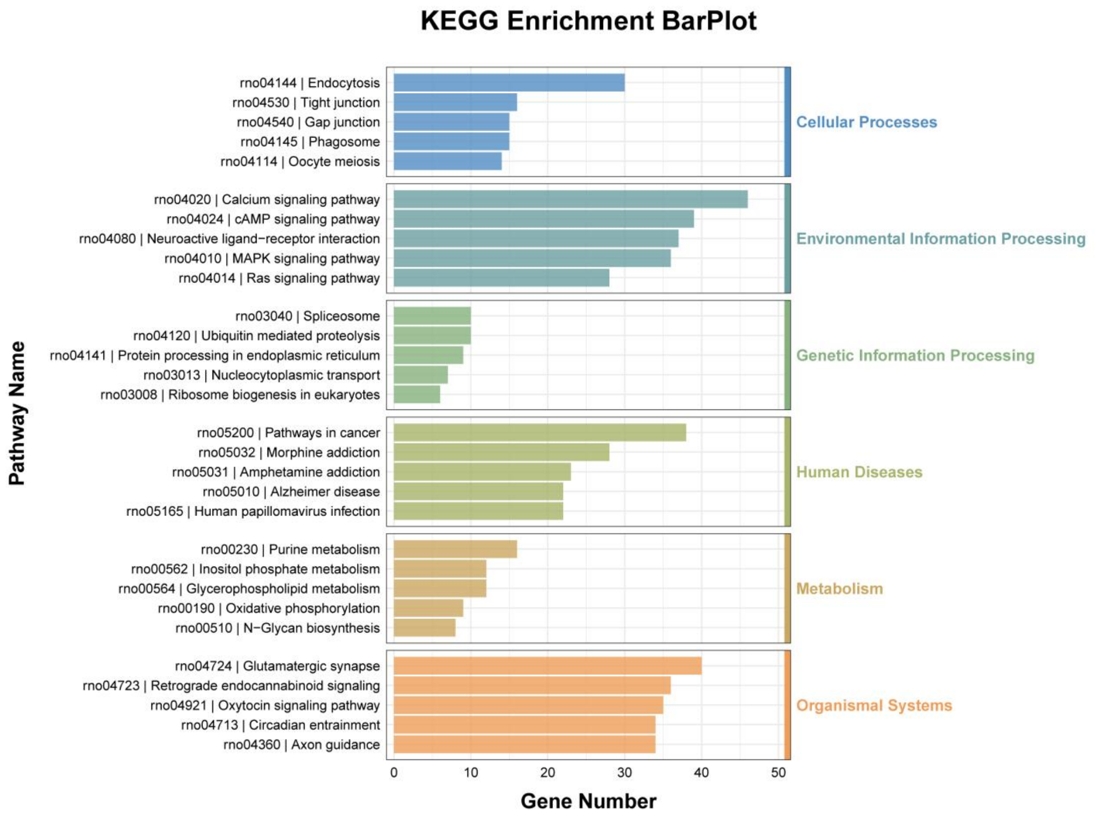


Supplementary Figure 6: KEGG enrichment analysis in the neuronal 4 clusters

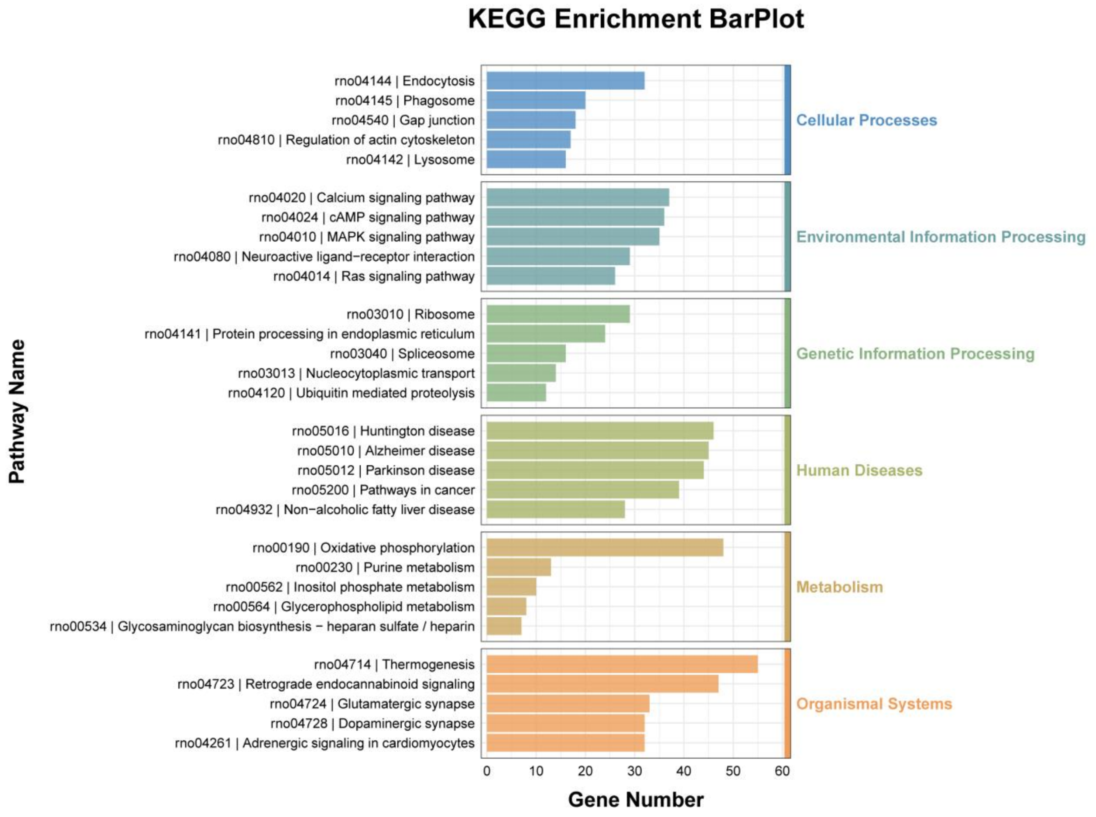


Supplementary Figure 7: KEGG enrichment analysis in the neuronal 5 clusters

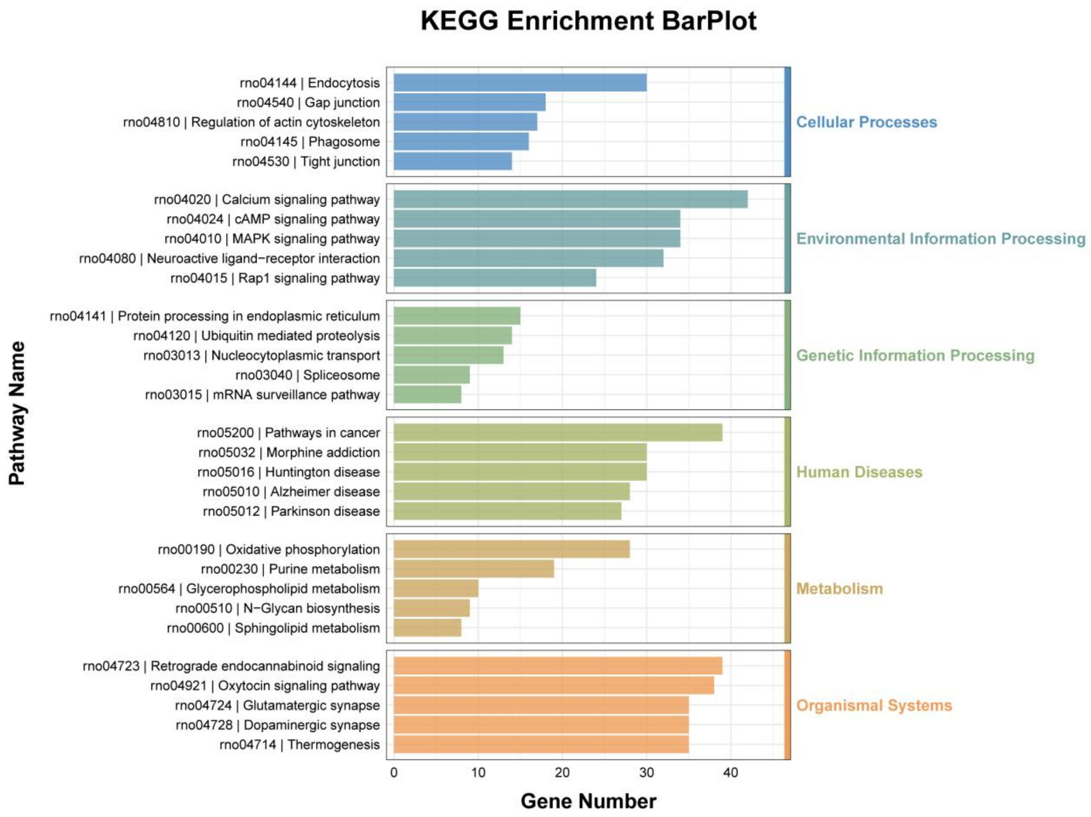


Supplementary Figure 8: KEGG enrichment analysis in the neuronal 14 clusters

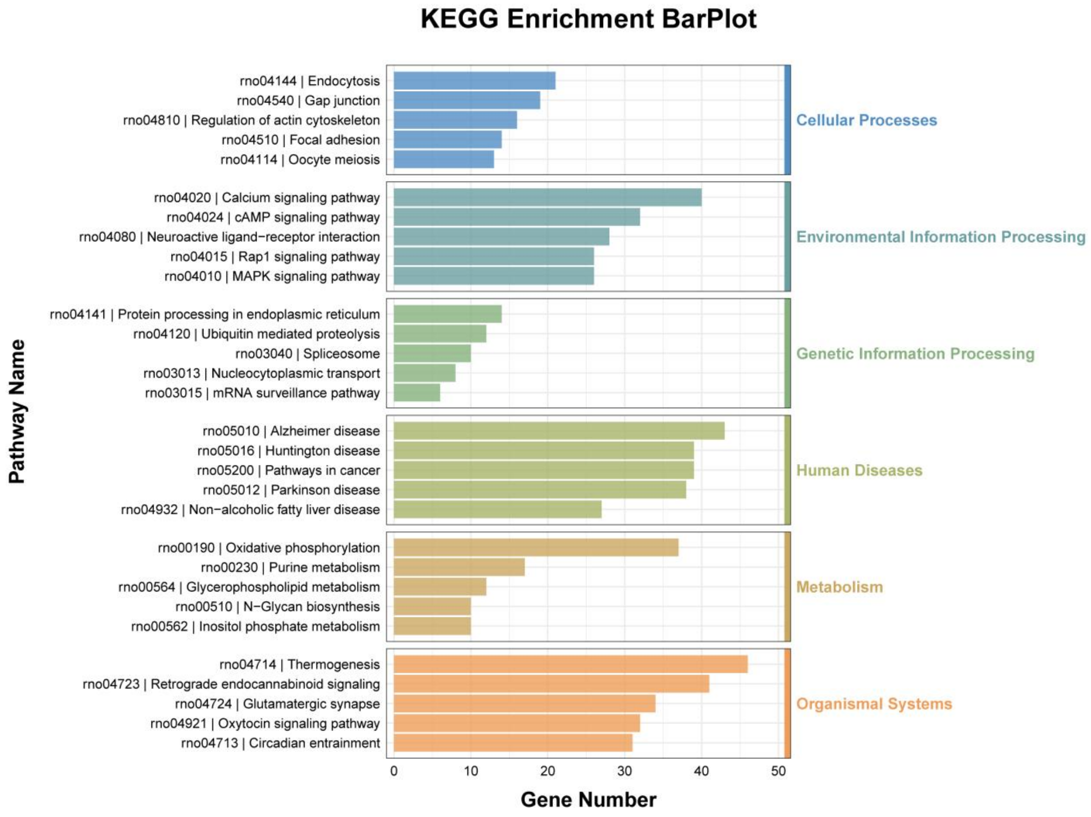


Supplementary Figure 9: KEGG enrichment analysis in the neuronal 15 clusters

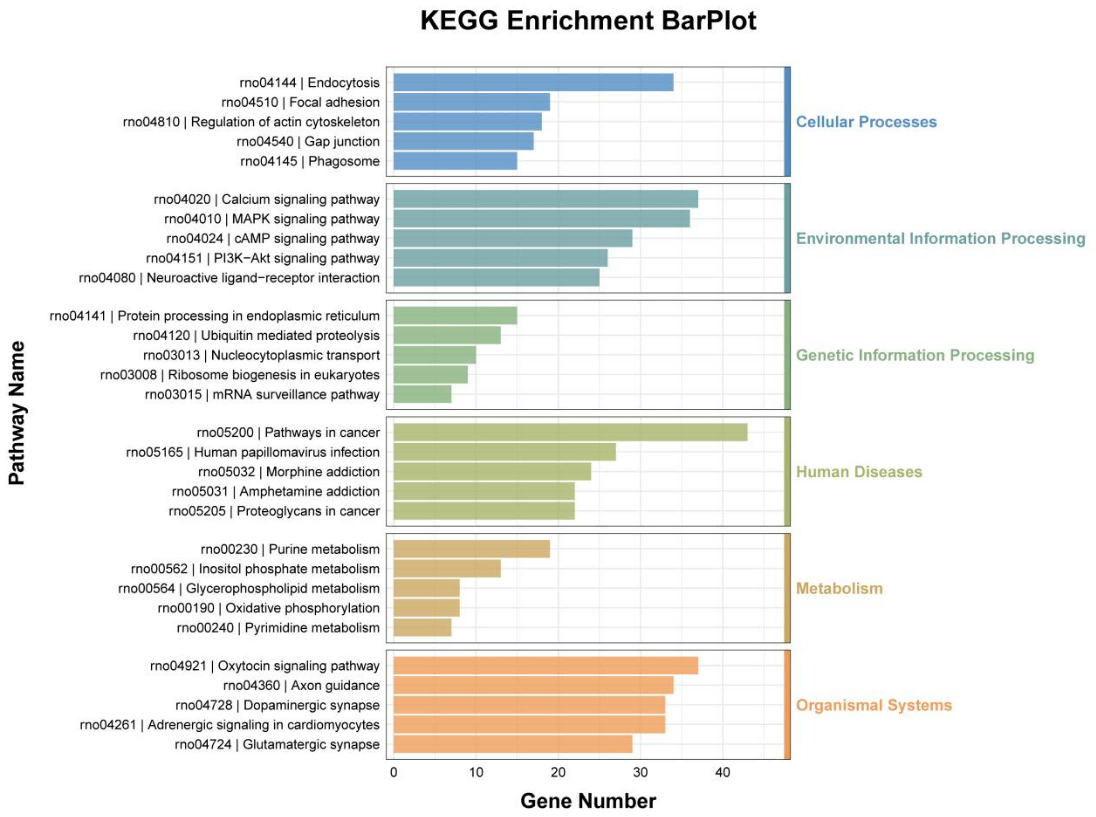


Supplementary Figure 10: KEGG enrichment analysis in the neuronal 17 clusters

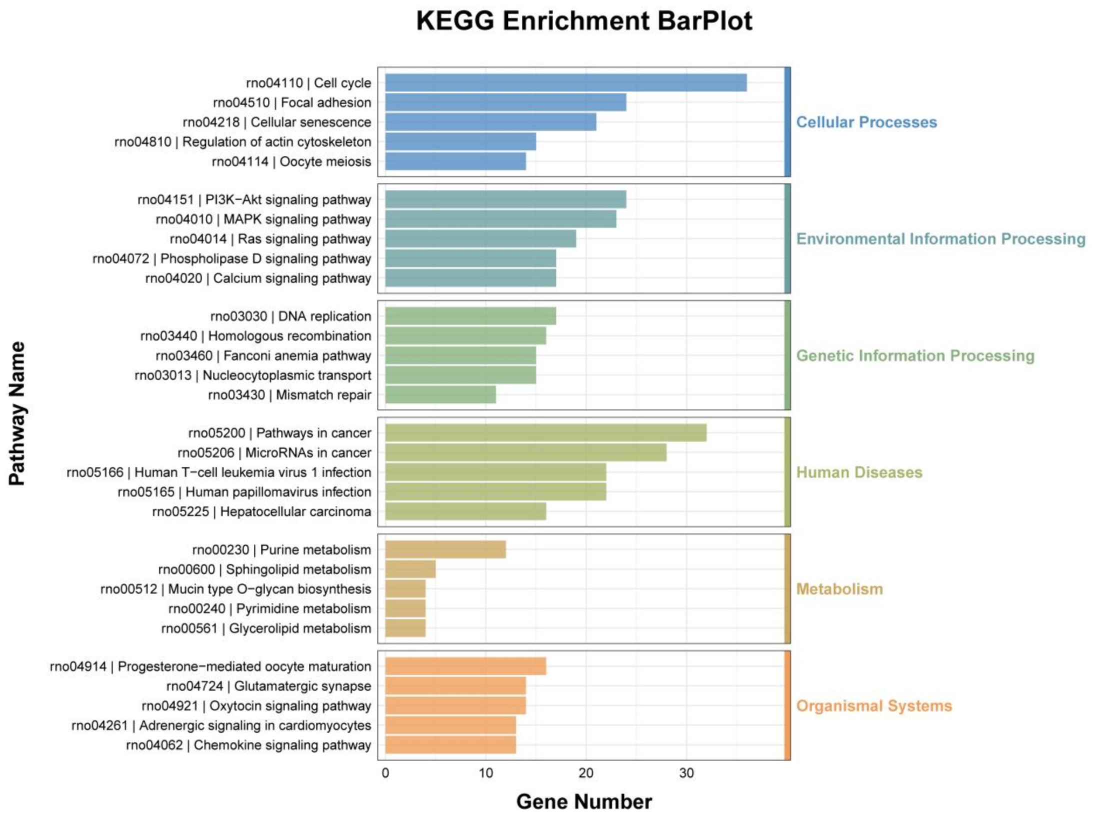


Supplementary Figure 11: KEGG enrichment analysis in the neuronal 20 clusters

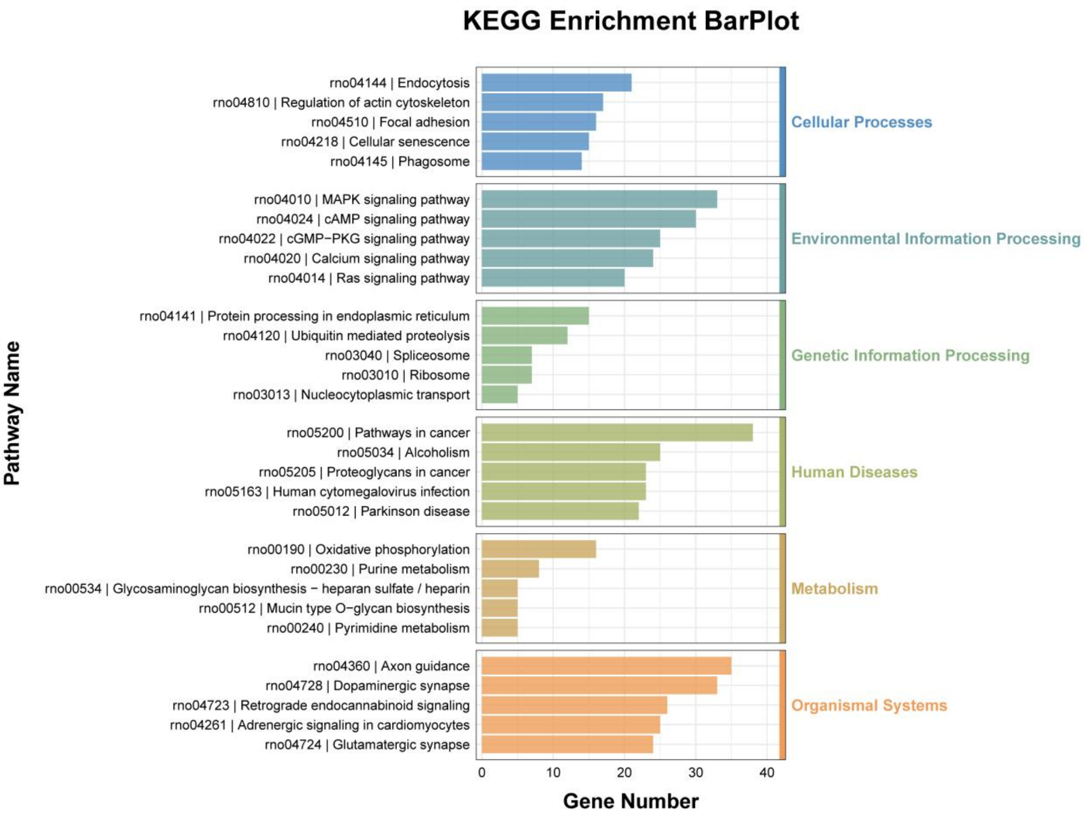


Supplementary Figure 12: KEGG enrichment analysis in the neuronal 21 clusters
